# Characterization of a Novel SOD-1(G93A) Transgenic Mouse Line with Very Decelerated Disease Development

**DOI:** 10.1371/journal.pone.0015445

**Published:** 2010-11-11

**Authors:** Alexandre Henriques, Claudia Pitzer, Armin Schneider

**Affiliations:** 1 Department of Molecular Neurology, SYGNIS Bioscience, Heidelberg, Germany; 2 INSERM U692, Strasbourg, France; 3 Faculty of Medicine, UMRS692, University of Strasbourg, Strasbourg, France; Julius-Maximilians-Universität Würzburg, Germany

## Abstract

Amyotrophic Lateral Sclerosis (ALS) is a fatal motoneuron disease, characterized by progressive weakness, muscle wasting and death ensuing 3–5 years after diagnosis. The etiology of ALS is complex and therapeutic approaches rely mostly on transgenic animal models with SOD-1 mutations. Most frequently employed is a mouse line transgenic for SOD-1 (SOD-1 Tg) that contains a point mutation at amino acid position 93 (G->A), present in patients suffering from a familial form of amyotrophic lateral sclerosis. Here we report on a SOD-1 (G93A) Tg mouse line with abnormally delayed onset of disease and prolonged survival. This phenotype arose spontaneously in our colony of the classic SOD-1 (G93A) line. We found that the copy number of the SOD-1 transgene was drastically decreased. We established a new breeding colony, the SOD-1 (G93A)^PS^ line (*PS* for *prolonged survival*) where the phenotype is stably inherited for 4 generations now. The mice develop symptoms at an age of approximately 12 months and die at 15 months of age. The delayed development of disease may more closely mimic human pathophysiology, and studying drug effects in this model may yield added confidence for potential efficacy of ALS drug candidates.

## Introduction

Amyotrophic Lateral Sclerfosis (ALS) is an incurable fatal motoneuron disease, characterized by progressive weakness, muscle wasting and death ensuing 3–5 years after diagnosis [1]. For the majority of the ALS cases, the disease etiology is sporadic, but ∼10% of ALS patients have a familial form of ALS. Most of the familial cases are due to mutations within the gene encoding the superoxide dismutase 1 (SOD-1) protein, an enzyme involved in the detoxification of reactive oxygen species [2]. Transgenic animals carrying mutations in the SOD-1 gene have been generated and they develop similar symptoms that what is observed in clinic [3]. Among the different animal models, the most studied one is a mouse line carrying a point mutation in amino acid position 93 of the SOD-1 protein (G ->A) that have a life expectancy of 150 days [4]. Previous reports indicate that slight spontaneous drops in transgene copy number result in a change in symptoms severity and disease progression [5]. Here, we report on a novel SOD-1 (G93A) transgenic mouse line with a drastically reduced copy number of the SOD-1 (G93A) transgene.

## Methods

### Maintenance of the SOD-1 (G93A) colony

Mice transgenic for the SOD1(G93A) mutation [3] on a C57BL/6 background (B6.Cg-Tg(SOD1-G93A)1Gur/J strain; Jackson laboratory, Bar Harbour, Maine) that harbor the high copy number of the mutant allele human SOD1 were maintained as a hemizygous line in an SPF-breeding facility (University of Heidelberg, Interfakultäre Biomedizinische Forschungseinrichtung (IBF)). The hemizygous line was maintained by mating transgenic males with C57BL/6 wild-type females.

### Assessment of disease progression

Body mass were recorded at regular interval at 9 am. Onset of weight decrease was defined as a drop of 5% of the mouse maximal weight. Muscular strength was followed by grip strength measurements (GS Columbus). Mean of three tests was recorded. Clinical onset of disease was defined as beginning of gait impairment, defined as abnormal limb movement in at least one hind limb. Clinical end stage was defined as inability of the animal to right itself over a period of 30 s. Animals were sacrificed at that point. Disease duration was defined as the time in day between the clinical onset of disease and the clinical end point of disease.

### Quantitative PCR

Copy numbers of the SOD-1 transgene were estimated by quantitative PCR on genomic DNA by comparing SOD-1/cyclophilin ratios of SOD-1 Tg mice and SOD-1 (G93A)^PS^ mice. The following primer sets were used: SOD-1^G93A^s (GTG TGC GTG CTG AAG GGC GA), SOD-1^G93A^as (CCA CCT TTG CCC AAG TCA TCT GC), cyc5 s (ACC CCA CCG TGT TCT TCG AC), cyc300as (CAT TTG CCA TGG ACA AGA TG), 60°C, 82°C. Amplification conditions were 60°C annealing and 81°C measuring temperature for the cyclophilin product, and 62°C annealing and 78°C measuring temperature for the SOD-1^G93A^ product. Specificity of product was ensured by melting curve analysis and agarose gel electrophoresis. Relative levels were derived after normalization to cyclophilin.

### Conduct of experiments and statistics

Experiments were performed in a blinded manner with blindness of all experimenters to genotype identity as long as possible. Group or pairwise parametric or non-parametric comparisons were done using JMP 8.01 (SAS Institute) or NCSS software (NCSS, Kaysville, Utah). Survival and onset data were analyzed using the logrank test. A p value of <0.05 was considered significant. All animal experiments were approved by the ethics committee of the Regierungspräsidium Karlsruhe, Germany (referat 35) with the ID approval number AZ 35-9185.81/G-151/06.

## Results

**Figure 1:**
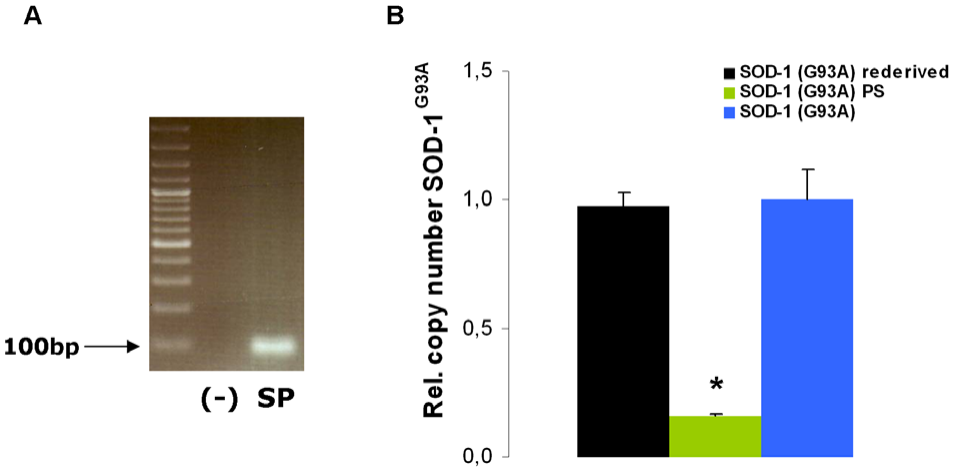
The SOD-1 (G93A) transgene in the SOD-1 (G93A)^PS^ line. (A) Genotyping of the SOD-1 (G93A)^PS^ mouse reveals a characteristic band from the SOD-1 (G93A) transgene. (B) Copy number of the SOD-1^G93A^ transgene is reduced in the SOD-1 (G93A)^PS^ mice by a factor of 6 when compared to normal SOD-1 (G93A) mice, littermates from the first SOD-1 (G93A)^PS^ mouse or from newly rederived SOD-1 (G93A) mice (p<0.05).

In our breeding colony of SOD-1 (G93A) transgenic mice we noted absence of typical ALS-like symptoms in a female mouse at a time point where mice from the original SOD-1 (G93A) line usually suffer from strong gait impairment and reduced motor function. Initially we suspected an error in genotyping. Genotyping was therefore repeated and confirmed that the mouse was carrying the SOD-1 transgene (Fig. 1a). We bred this mouse to wild type C57BL/6 mice, and were able to preserve the phenotype in about 50% of the offspring, consistent with a Mendelian inheritance pattern. We refer to this mouse and its offspring as the SOD-1 (G93A)^PS^ (for *prolonged survival*) line.

We determined transgene copy number by quantitative PCR on genomic DNA. The SOD-1 (G93A)^PS^ line showed a drastic (about six-fold) drop in their copy number of the SOD-1 transgene when compared to normal SOD-1 Tg mice (Fig. 1b). The copy number was however stable within subsequent generations of the SOD-1 (G93A)^PS^ line. According to previous quantification of transgene copy number [3], [6], the SOD-1 (G93A) mice harbours 25 copy of the SOD-1 (G93A) transgene. We deduce from the PCR quantification that the SOD-1 (G93A)^PS^ line harbours 4 copies of the transgene.

**Figure 2:**
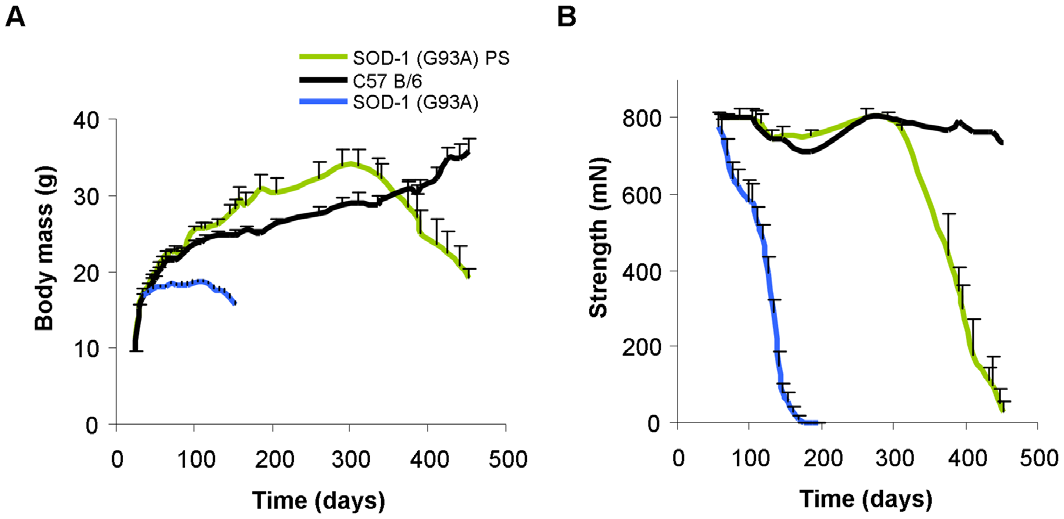
Weight loss and muscular strength deficit are delayed in the SOD-1 PS line. (A) The SOD-1 (G93A)^PS^ mice (n = 8) have a delayed onset of weight decrease when compared to the original line (n = 17) (p<0.05). (B) Normal SOD-1 Tg mice start with a decrease in muscle strength at the age of 70 days. SOD-1 (G93A)^PS^ mice have normal muscular strength until the age of 310 days (p<0.05).

We then systematically studied the phenotype by monitoring muscular strength, body weight, symptoms progression and survival (n = 8 mice per group (wt littermates, and SOD-1 (G93A)^PS^ line), n = 17 mice from original SOD-1 (G93A) line, all female)). There are obvious differences in the development of disease symptoms in the SOD-1 (G93A)^PS^ line when compared to the original line. In the SOD-1 (G93A)^PS^ line, the muscular deficit and body weight decrease occur later than in the original line (p<0.05, Fig. 2). Surprisingly, we noted that during the presymptomatic phase mice from the SOD-1 (G93A)^PS^ line have a relative increase in body mass compared to the wild type littermates (5 g maximal difference, p<0.05). This apparently counterintuitive finding merits further exploration particularly in view of reports on metabolic disturbances in mutant SOD-1 transgenic mice. Interestingly, higher food intake was observed in presymptomatic mice of the SOD-1 (G86R) transgenic line [7].

**Figure 3:**
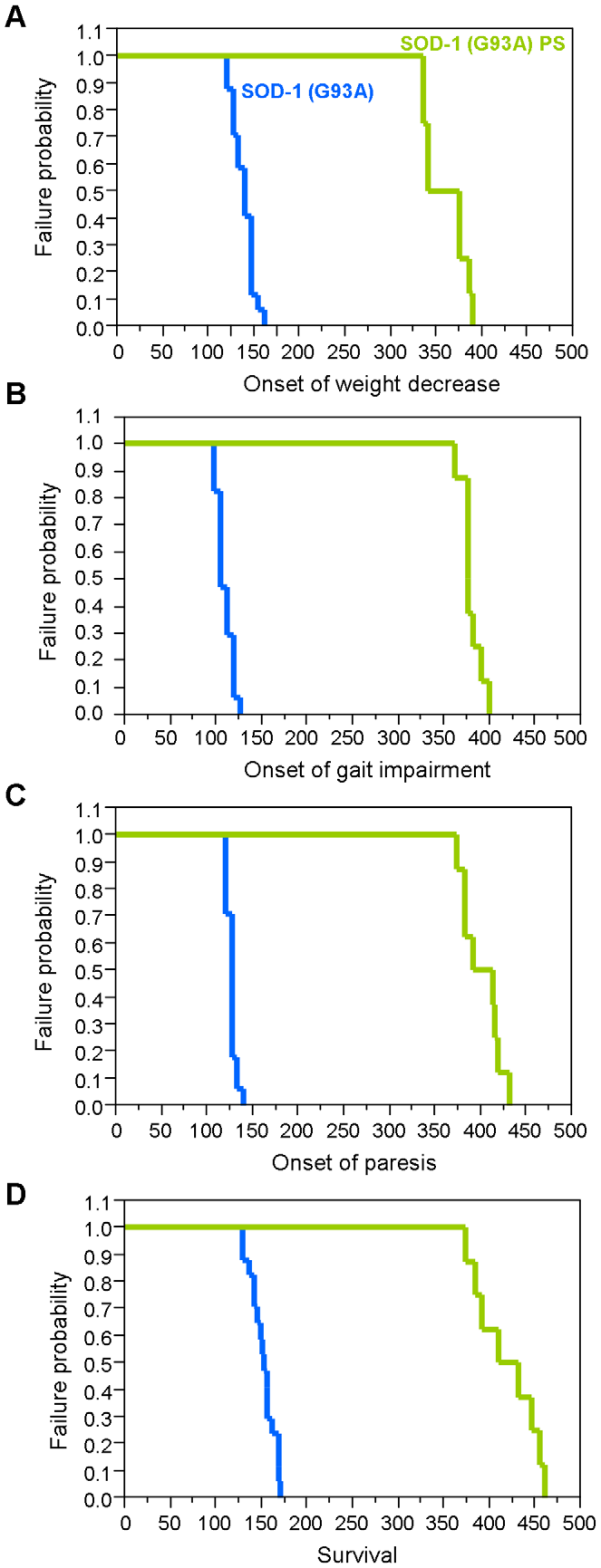
Timed data for onset and survival. Given are parameters for disease onset (A-C), and survival (D) for the SOD-1 (G93A) (n = 17) and SOD-1 (G93A)^PS^ mice (n = 8). (A) Onset of weight decrease was defined as a drop of 5% of the mouse maximal weight. (B) First manifestation of symptoms that are linked to gait impairment in terms of limbs coordination and overall stance, and (C) time to first manifestation of paresis. (D) End point of the disease, defined as the inability of the mouse to right itself within 30 seconds.

Regarding the clinical symptoms, substantial differences have been observed as well (Fig. 3). In the original line, mice develop tremor in the hindlimbs around 70 day of age, gait impairment around the age of 110 days, and reach the endpoint of disease at 150 day of age. In the SOD-1 (G93A)^PS^ line, tremor was inconsistent and not present in all mice. Gait impairment was however consistently present and first observable at 380 days of age. Mice reached the clinical end point of the disease at 418 days of age (Table 1). We monitored survival through 4 subsequent generations and found that the life expectancy was equal, suggesting that the new line is genetically and phenotypically stable (Table 2). As tremor is not a reliable symptom in the new line, we defined the disease onset as the first occurrence of gait impairment at 380 days of age. It is interesting to note that the disease duration (time between disease onset and disease end point) is about 42 days and therefore similar between the original and the *PS* line (Table 1).

**Table 1:** Listed are key characteristics of ALS-like symptoms in the original SOD-1 (G93A) and in the new SOD-1 (G93A)^PS^ line (mean±SEM).

	SOD-1 G93A	SOD-1 G93A ^PS^	p value
**Weight decrease (days)**	138.35±3.0	359.4±8.5	<0.05
**Onset of gait impairment (days)**	109.5±2.1	380.3±4.0	<0.05
**Survival (days)**	151.5±5.1	418.6±11.9	<0.05
**Disease duration (days)**	42.0±4.8	41.22±9.9	>0.60

**Table 2:** Listed are survival times (mean±SEM) in the SOD-1 (G93A)^PS^ line through 4 subsequent generations.

SOD-1 G93A ^PS^ generation	F0	F1	F2	F3
**Survival (days)**	444	436	418.6±11.9	421.4±15.5
**Mice per group**	1	1	8	9

Survival times are highly similar suggesting that the phenotype of the new line is stable.

We conclude that the drop in transgene copy number in our new line led to strong delay of disease onset and extended survival, but does not prolong disease duration.

## Discussion

SOD-1 (G93A) transgenic mouse lines with low copy numbers have been previously described by other groups. The first line reported is almost as old as the original line [8]. These mice harbour around 14 to 17 copies of the SOD-1 (G93A) transgene [9], have a prolonged asymptomatic phase, and a reported survival between 200 days [10] and 273 days, with a disease duration (disease onset defined as apparition of motor impairment) of 49 days [11]. The disease duration is comparable to what we report here (see Table 1). The line described here has however the longest disease-free interval, the lowest copy number, and longest survival of all reported lines.

The similar disease duration between the mouse lines is interesting because it suggests that the transgene number copy has an influence on the length of the asymptomatic phase but not on the disease duration. This implies that once the onset of disease is reached, the disease starts and progresses at a precise pace. Mouse lines with low transgene copy numbers are therefore interesting for the study of the asymptomatic phase and factors that determine disease onset.

The fact that dramatic losses of transgene copies occur in the SOD1 (G93A) line even after a long time of breeding brings one issue to mind that is important in pre-clinical drug studies: Control of copy number. Recently, doubts have been raised as to the relevance of the SOD-1^G93A^ mouse line as an animal model of ALS. These doubts are based on the clinical phase III failure of several compounds that had beneficial effects in the SOD-1 (G93A) Tg line [12]. Scott and colleagues point out several problematic points with the SOD1 line [13]. Slight variations in transgene copy numbers is one of the explanations given for potentially erroneous results in animal studies, as survival correlates inversely with the copy number [5]. However, a drop in copy number is judged to be a rare event, with likely moderate influence on preclinical results. Apart from small copy number variations, major losses of copy numbers can happen even after the long maintenance of the Guerney line. These alterations may propagate in a colony of transgenic animals if undetected. We strongly recommend to quantify the copy number for all SOD-1 Tg animals used for breeding and experiments.

Here, we report a mouse line with drastically reduced copy number of the SOD-1 (G93A) transgene. The mice of this line exhibit a long asymptomatic phase, reminiscent of the disease course in ALS patients. Also, SOD-1 (G93A)^PS^ mice develop the phenotype at an advanced age that may be more comparable to human patients where the disease rarely strikes below 40, and is most commonly seen around the age of 60. Therefore this novel line may provide a useful additional model for establishing proof-of-concept for new therapeutic concepts.

As the presymptomatic phase is longer, the SOD-1 (G93A)^PS^ mice may also be useful for investigations into the pathophysiological mechanisms taking place before the apparition of clinical symptoms. Finally, the mice described here may be especially valuable for testing preventive strategies.
